# Genotyping and Molecular Diagnosis of Hepatitis A Virus in Human Clinical Samples Using Multiplex PCR-Based Next-Generation Sequencing

**DOI:** 10.3390/microorganisms10010100

**Published:** 2022-01-04

**Authors:** Geum-Young Lee, Won-Keun Kim, Seungchan Cho, Kyungmin Park, Jongwoo Kim, Seung-Ho Lee, Jingyeong Lee, Young-Sun Lee, Ji Hoon Kim, Kwan Soo Byun, Jin-Won Song

**Affiliations:** 1 Department of Microbiology, Korea University College of Medicine, Seoul 02842, Korea; gemyeng002@korea.ac.kr (G.-Y.L.); schanchan@korea.ac.kr (S.C.); kmpark0131@korea.ac.kr (K.P.); hotdog442@korea.ac.kr (J.K.); leeds1104@korea.ac.kr (S.-H.L.); yoj0702@korea.ac.kr (J.L.); 2 Department of Microbiology, College of Medicine, Hallym University, Chuncheon 24252, Korea; wkkim1061@hallym.ac.kr; 3 Institute of Medical Science, College of Medicine, Hallym University, Chuncheon 24252, Korea; 4 BK21 Graduate Program, Department of Biomedical Sciences, Korea University College of Medicine, Seoul 02842, Korea; 5 Division of Gastroenterology and Hepatology, Department of Internal Medicine, Korea University Medical Center, Seoul 08308, Korea; lys810@korea.ac.kr (Y.-S.L.); kjhhepar@naver.com (J.H.K.); kwsbyun@unitel.co.kr (K.S.B.)

**Keywords:** hepatitis A virus, multiplex polymerase chain reaction, next-generation sequencing, phylogenetic analysis, genotypic analysis

## Abstract

Hepatitis A virus (HAV) is a serious threat to public health worldwide. We used multiplex polymerase chain reaction (PCR)-based next-generation sequencing (NGS) to derive information on viral genetic diversity and conduct precise phylogenetic analysis. Four HAV genome sequences were obtained using multiplex PCR-based NGS. HAV whole-genome sequence of one sample was obtained by conventional Sanger sequencing. The HAV strains demonstrated a geographic cluster with sub-genotype IA strains in the Republic of Korea. The phylogenetic pattern of HAV viral protein (VP) 3 region showed no phylogenetic conflict between the whole-genome and partial-genome sequences. The VP3 region in serum and stool samples showed sensitive detection of HAV with differences of quantification that did not exceed <10 copies/μL than the consensus VP4 region using quantitative PCR (qPCR). In conclusion, multiplex PCR-based NGS was implemented to define HAV genotypes using nearly whole-genome sequences obtained directly from hepatitis A patients. The VP3 region might be a potential candidate for tracking the genotypic origin of emerging HAV outbreaks. VP3-specific qPCR was developed for the molecular diagnosis of HAV infection. This study may be useful to predict for the disease management and subsequent development of hepatitis A infection at high risk of severe illness.

## 1. Introduction

Hepatitis A virus (HAV) infections are the critical etiology of viral hepatitis and impose a massive socioeconomic burden worldwide [1]. HAV is mainly transmitted via the fecal-oral route through contaminated food and water. The prevalence rate of HAV in different countries varies with hygiene levels, and approximately, 1.5 million people are annually infected with HAV worldwide. In the late 1970s and early 1990s, 85–95% of the population were serologically positive for anti-HAV immunoglobulin in Republic of Korea (ROK), China, Japan, Philippines, and Thailand [2]. The seroprevalence of anti-HAV antibodies in the Korean population rapidly declined from more than 80% in the 1970s to less than 20% in 2007 [3,4]. The Korea Disease Control and Prevention Agency received approximately 250,379 reports of HAV infections in 2002–2019. Two endemic outbreaks of HAV occurred in 2009 and 2019, with 54,576 and 17,635 cases, respectively. While most HAV-infected children are asymptomatic, older adults with HAV infections exhibit a broad range of clinical manifestations such as anicteric infection and severe fulminant hepatic failure.

HAV (genus, *Hepatovirus*; family, *Picornaviridae*) is a non-enveloped virus with a single-stranded 7.5 kb genome that contains 5’ and 3’ noncoding regions, one open reading frame (ORF), and a poly(A) tract [5]. The 5’ untranslated region (UTR) of the HAV RNA harbors an internal ribosome entry site, which directs the ribosomes to the initiation codon of the polyprotein. The single ORF is divided into three functional regions: the P1 region that encodes the capsid polypeptides viral protein 1 (VP1) to VP4 and the P2 (2A, 2B, and 2C) and P3 (3A, 3B, 3C, and 3D) regions that encode nonstructural polypeptides associated with viral replication. Globally spread HAV strains display significant variability in RNA genomic sequences, despite the limited heterogeneity of amino acids [6]. In 1987, a genetic analysis of HAV strains was conducted for seven genotypes including human (I–III and VII) and simian (IV–VI) groups. The HAV strains have six sub-genotypes (IA, IB, IIA, IIB, IIIA, and IIIB) in humans. Phylogenetic and genotypic analyses of HAV were performed using partial-genome sequences, including the VP1/P2A junction [5,7], VP1/P2B junction [8,9], C termini of VP3 [7], N terminus of VP1 [8,10], entire VP1 [11,12], and 5’ UTR [13] regions. In clinical microbiology, the partial-genome sequences of HAV exhibited inaccurate assessment of phylogeographic relationships or potential transmission routes in an outbreak [14]. Therefore, the precise genotypic relationship of global HAV strains with partial-genome sequences remains to be investigated.

Genomic epidemiology of HAV plays a critical role in identifying the epidemiologic surveillance, microbial source tracking, and pathogen identification of food-borne outbreak [4]. The genomic epidemiology of emerging HAV in eastern Spain demonstrated a food-borne origin of the virus that caused the outbreak and patients with hepatitis A [15,16]. Epidemiological analysis of tourists returning from Egypt identified that orange juice was the source of infection in the hepatitis A outbreak [17]. Genomic epidemiology of HAV IB genotype reported the causes of an outbreak associated with homelessness and drug abuse in USA [14]. Epidemiologic investigations of hepatitis A outbreaks revealed that hepatitis A cases had consumed semi-dried tomatoes in Australia, Netherlands, and England [18,19,20]. However, identification and sequencing of HAV remains challenging due to the low titer of pathogens in human or food samples.

Next-generation sequencing (NGS) has a broad range of applications, including simultaneous multiple pathogens detection, phylogenetic diversity estimation, and whole-genome sequencing (WGS) [21,22,23,24,25,26,27,28]. Metagenomic NGS combined with spiked primer enrichment and/or tiling multiplex PCR recovered viral genomes from coronavirus disease 19 patients, revealing the genetic diversity of severe acute respiratory syndrome coronavirus 2 (SARS-CoV-2) and the transmission of virus lineages [29]. Multiplex polymerase chain reaction (PCR)-based NGS acquired the nearly WGS of Hantaan virus (HTNV) directly from hemorrhagic fever with renal syndrome (HFRS) patient samples and rodent tissues [27]. These studies demonstrated the putative infection site of HTNV by phylogeographic analysis in HFRS-endemic areas. NGS allowed nearly WGS of HAV in frozen berries linked to HAV outbreaks in Italy [30].

In this study, multiplex PCR-based NGS facilitated nearly WGS of HAV directly from samples collected from patients with hepatitis A. The results demonstrated that HAV VP3 gene might be a potential candidate for tracing the genotypic origin of an outbreak, showing a well-established phylogenic pattern compared with the whole-genome sequences of HAV. Overall, our findings provide significant insights into the genomic epidemiology, genetic identification, and molecular diagnosis of hepatitis A infection. 

## 2. Materials and Methods

### 2.1. Ethics Approval and Participation Consent

Written informed consent was obtained from all participants, and the Institutional Review Board of the Korea University Guro Hospital (2008GR0015) approved this study.

### 2.2. Study Population and Sample Collection

Clinical samples were obtained from the Korea University Guro Hospital, Seoul, Korea. Hepatitis A infection was diagnosed based on laboratory and clinical tests in five patients. Serum and stool samples from patients with anti-HAV immunoglobulin M (IgM) antibodies were used in this study. All clinical samples were collected from patients of 22–38 years in age.

### 2.3. Reverse Transcription-PCR (RT-PCR) Assay

Total RNA was extracted using TRI Reagent LS (Ambion, Austin, TX, USA). Reverse transcription was performed using 1 µg of RNA with a High-Capacity RNA-to-cDNA kit (Applied Biosystems, Foster City, CA, USA) and random hexamers. Primer sequences used for RT-PCR included HAV VP3-F1 (forward), 5′-GCT TTG GAT CAG GAA GAT TGG A-3′; HAV VP3-F2 (forward), 5′-ACT CAT TTT ACT TUT TTG GAC ATC CA-3′; and HAV VP3-R1 (reverse), 5′-CAT GAT AAA GAG GAG CAA AAC ATT CC-3′. The first and second RT-PCRs were performed at 94 °C for 5 min, followed by six cycles at 94 °C for 40 s, 40–45 °C for 1 min, and 72 °C for 1–2 min; 32 cycles at 94 °C for 40 s, 42 °C for 40 s, and 72 °C for 1–2 min; and a final cycle at 72 °C for 5 min (ProFlex PCR System, Life Technology, CA, USA). 

### 2.4. Quantitative PCR (qPCR) Assay

The recombinant plasmid DNA of HAV VP3 gene was isolated using the pTOP Blunt V2 vector (Enzynomics Co., Ltd., Seoul, Korea). The concentration of recombinant plasmid DNA was measured by UV absorbance at 260 nm and 280 nm using Nano drop (Thermo Fisher Scientific, CA, USA). Standard curve was obtained from serial 10-fold dilutions of recombinant plasmid DNA ranging from 1 × 10^10^ to 1 × 10 copies/µL. The copy number of plasmids per microgram of DNA was calculated using the total number of nucleotides in the plasmid using a previously described formula [31]. The HAV-specific primer set was designed based on the VP3 region: HAV VP3-qF (forward), 5′-ATG AAG ATG CAA GGG CAA A-3′ and HAV VP3-qR (reverse), 5′-GGA ATG GAT GTC CAA GTA GTA AA-3′. Viral RNA quantification was compared with the qPCR assay of the VP4 genomic region targeting the HAV genotype IA [32]. qPCR was performed at 95 °C for 10 min, followed by 40 cycles at 95 °C for 15 s and 60 °C for 1 min using a QuantStudio 5 Real-Time PCR System (Applied Biosystems, Foster City, CA, USA) with a Power SYBR Green PCR Master Mix (Applied Biosystems, Foster City, CA, USA). 

### 2.5. Multiplex PCR-Based Next-Generation Sequencing

For the whole-genome strategy, we used several sets of primers to amplify overlapping fragments of 200–500 bp in length spanning the entire viral genome (Table S1). The cDNA library was enriched using Solg 2× Uh-Taq PCR Smart mix (SolGent, Daejeon, Korea), according to the manufacturer’s instructions. The reaction mixture of 25 μL contained 12.5 μL of 2× Uh pre-mix, 2 μL of each primer, 9.5 μL of distilled water, and 1 μL of DNA template. Multiplex PCR was performed at 95 °C for 15 min, followed by 40 and/or 25 cycles at 95 °C for 20 s, 50 °C for 40 s, 72 °C for 1 min, and a final cycle at 72 °C for 3 min. 

DNA libraries were prepared using a TruSeq Nano DNA LT Sample Preparation Kit (Illumina, San Diego, CA, USA), according to the manufacturer’s instructions. To obtain size-selected amplicons, cDNA templates were mechanically sheared using an M220 focused ultrasonicator (Covaris, Woburn, MA, USA). The cDNA amplicons were prepared by size selection, A-tailing, and ligation with indexes and adaptors. The enrichment reaction contained 5 μL of PCR primers and 20 μL of the enhanced PCR mixture (Illumina, San Diego, CA, USA). Library quality was evaluated using a bioanalyzer with an Agilent DNA 1000 Kit (Agilent Technologies, Santa Clara, CA, USA). NGS was performed using a 2 × 150 bp MiSeq benchtop sequencer (Illumina, San Diego, CA, USA) with an MiSeq Reagent Kit V2 (Illumina, San Diego, CA, USA).

### 2.6. Phylogenetic Analysis

Whole-genome sequences of HAV were aligned using ClustalW in Lasergene 5 (DNASTAR, Madison, WI, USA). The phylogenetic tree was generated using the best-fit general time reversible+gamma+invariable model of evolution. Support for topologies was assessed using bootstrapping for 1000 iterations. After model optimization for each data set, the pairwise genetic distance between HAV strains was calculated using MEGA 7.0 [33]. 

### 2.7. Tanglegram Analysis for Comparing Different Phylogenies

To compare the phylogenetic patterns in the whole-genome and partial-genome sequences of HAV, two phylogenetic trees were generated using the tanglegram algorithm for matching HAV strains. The auxiliary lines in the center connected the phylogenetic trees. Tanglegrams were generated for all phylogenetic links between the whole-genome and partial-genome sequences using the ‘dendextend’ package of R [34].

## 3. Results

### 3.1. Clinical Symptoms of HAV-Infected Patients

Five patients exhibited symptoms of acute hepatitis A (Table 1). All patients who tested positive for anti-HAV IgM showed significant elevation in aspartate aminotransferase, alanine aminotransferase, and alkaline phosphatase. All patients manifested typical symptoms compatible with acute viral hepatitis A (e.g., icterus, dark urine, anorexia, and malaise). All five patients manifested hyperbilirubinemia (serum bilirubin levels > 1.2 mg/dl). None of the participants had other chronic liver diseases such as chronic hepatitis B, chronic hepatitis C, autoimmune hepatitis, or primary biliary cholangitis. 

### 3.2. Molecular Diagnosis and Quantification of HAV Genomes

**Figure 1:**
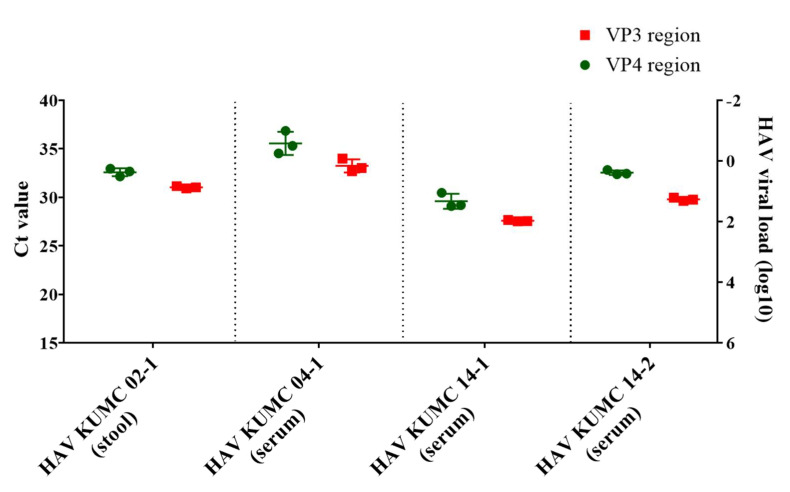
Quantitation of hepatitis A virus (HAV) RNA genome in clinical samples from patients in the Republic of Korea. Real-time quantitative polymerase chain reaction assay was performed for molecular diagnosis and quantification of HAV genomes in serum and stool samples. The vertical axis shows the HAV RNA copy numbers of VP4 and VP3 regions. Each data point represents the mean threshold cycle (Ct) value obtained from triplicates.

The viral load of the four serum and stool samples was quantified by HAV VP3-specific qPCR (Figure 1). The copy number of HAV RNAs was determined using a linear regression curve with a coefficient of correlation (r) value of 0.993. HAV KUMC 02-1, HAV KUMC 14-1, and HAV KUMC 14-2 showed Ct values of 27.6–31, corresponding to 10^1^–10^2^ viral RNA copies/μL in HAV-positive clinical samples, whereas HAV KUMC 04-1 showed a Ct value of 33, corresponding to <10 copies/μL of viral RNA. The threshold values of the HAV VP4 region were assessed at 29.2–35.3 with <10^2^ copies/µL of HAV RNA. 

### 3.3. Whole-Genome Sequencing and Genetic Analysis of HAV by Multiplex PCR-Based NGS

The coverage of HAV genomic sequences was associated with the viral RNA copy number (Table 2). Nearly whole-genome sequences of HAV (92.3–94.6%) were recovered from HAV KUMC 14-1, HAV KUMC 14-2, and HAV KUMC 02-1 that contained 10–100 viral RNA copies/μL. The HAV coverage rate was relatively low (86.1%) in HAV KUMC 04-1, as it contained only <10 viral RNA copies/μL. HAV KUMC 98-34 was excluded due to insufficient volume, and the whole-genome sequence was obtained by conventional Sanger sequencing.

Newly obtained HAV genome sequences were compared with representative HAV sequences of different sub-genotypes available in the GenBank (Table 3). HAV strains in ROK showed nucleotide and amino acid similarities with representative HAV strains of the sub-genotype IA at a rate of 93.8–99.8% and 97.1–99.7%, respectively. 

### 3.4. Sequence Similarity of HAV Genomes at the Genotype Level

Partial-genome sequences of the 52 HAV strains were assessed for gene homologies (Figure S1). The whole-genome (1–7477 nt) showed nucleotide similarities of 81.5–86.4%. The VP0 (VP4/VP2) (735–1469 nt), VP3 (1470–2207 nt), and VP1 (2208–3029 nt) regions showed sequence similarities of 83.1–87.7%, 82.7–87.2%, and 80.7–86.9%, respectively. The 2A (3030–3242 nt), 2B (3243–3995 nt), 2C (3996–5000 nt), and 2BC (3243–5000 nt) regions showed nucleotide similarities of 76.1–86.9%, 75.7–87.1%, 80.3–86.5%, and 78.6–86.3%, respectively. The 3A (5001–5222 nt), 3B (5223–5291 nt), 3AB (5001–5291 nt), 3C (5292–5948 nt), 3D (5949–7415 nt), and 3CD (5292–7415 nt) regions showed nucleotide similarities of 73.4–88.3%, 76.8–87%, 74.2–86.3%, 82.3–87.4%, 77.7–85.7%, and 79.7–86.3%, respectively. The VP1/P2A (2984–3217 nt), VP1/P2B (2896–3289 nt), and entire VP1 (2172–3125 nt) regions showed nucleotide similarities of 77.4–85.9%, 79.2–86.3%, and 78.9–87.2%, respectively.

### 3.5. Phylogenetic Analysis of Whole-Genome and Partial-Genome Sequences of HAV

**Figure 2:**
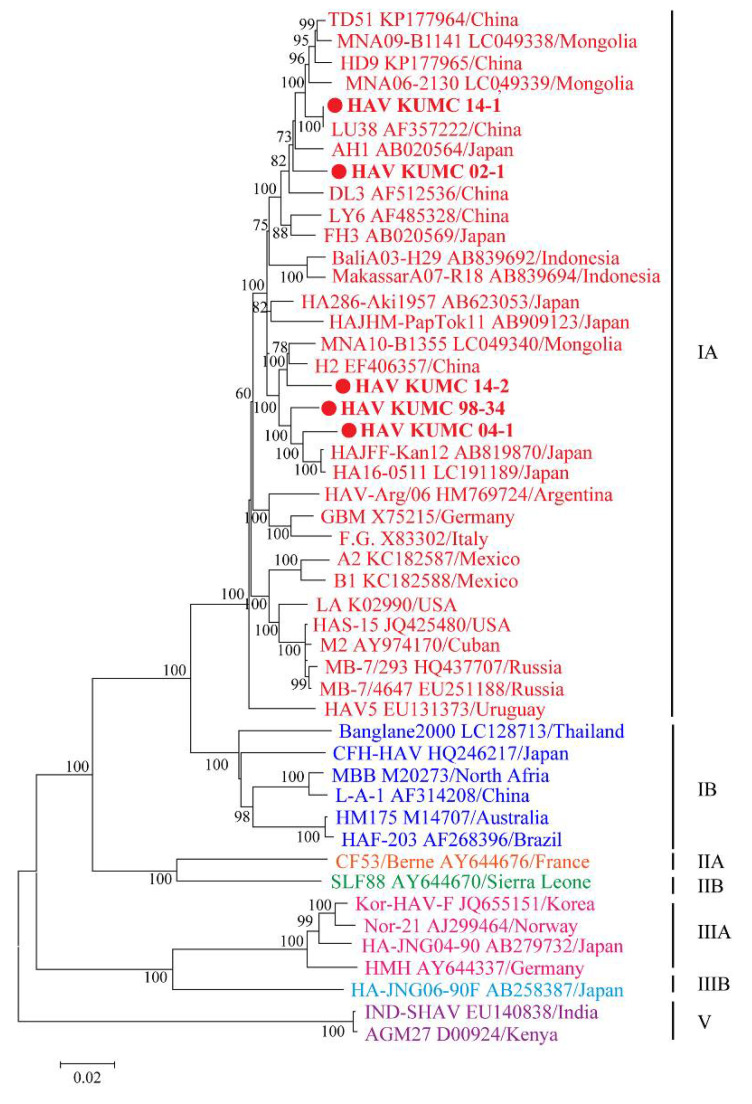
Phylogenetic analysis of the nearly whole-genome hepatitis A virus (HAV) sequences in the Republic of Korea (ROK). Nearly whole-genome sequences of HAV from serum and stool samples were obtained by multiplex polymerase chain reaction-based next-generation sequencing. Branch lengths are proportional to the number of nucleotide substitutions. Vertical distances are included for clarity. Phylogenetic trees of HAV were generated by the maximum likelihood method, with 1000 bootstrap iterations. Colored circles indicate specific HAV strains from ROK; red, genotype IA; blue, genotype IB; orange, genotype IIA; green, genotype IIB; pink, genotype IIIA; sky blue, genotype IIIB; and violet, genotype V.

The five HAV strains from ROK demonstrated a geographic cluster with the sub-genotype IA strains from Japan, China, and Mongolia (Figure 2). HAV KUMC 98-34 and HAV KUMC 04-1 formed a homologous genetic lineage with HAJFF-Kan12 and HA16-0511 from Japan. HAV KUMC 14-1 was phylogenetically grouped with LU38 from China, forming a distinct monophyletic branch with TD51, HD9, MNA09-B1141, and MNA06-2130 from China and Mongolia. HAV KUMC 02-1 was closely associated with HAV strains from Japan, China, and Mongolia, whereas HAV KUMC 14-2 showed the closest relationship with H2 and MNA10-B1355 from China and Mongolia.

**Figure 3:**
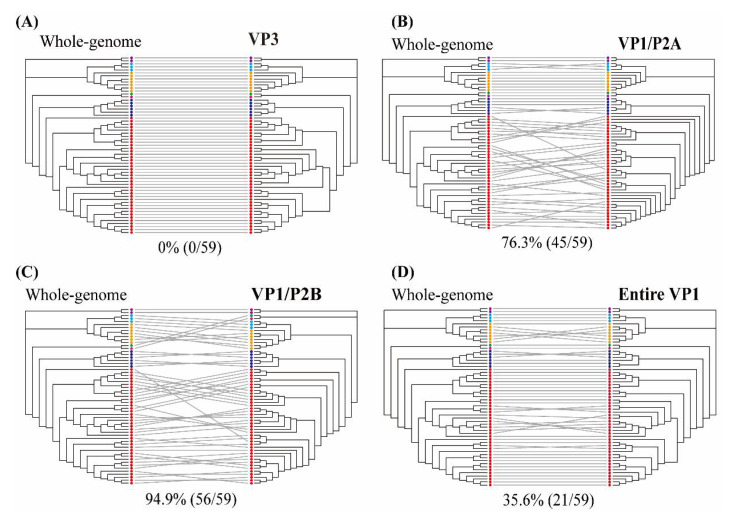
Tanglegram of hepatitis A virus (HAV) nucleotide sequences based on the whole- (**left**) and partial- (**right**) genome phylogenies. (**A**) VP3 region (1470–2207 nt), (**B**) VP1/P2A region (2984–3217 nt), (**C**) VP1/P2B region (2896–3289 nt), and (**D**) entire VP1 region (2172–3125 nt). Color indicates individual six sub-genotypes of human and one of sub-genotype of animal in HAV strains. Whole-genome and partial-genome phylogenies were generated using the maximum likelihood method. Full lines indicate significant phylogenetic conflicts. Grey lines indicate phylogenetic clade patterns falling between two different regions.

The individual HAV genes were estimated as the probability of intersecting clades compared with the phylogenetic pattern of whole-genome sequences (Figure 3 and Figure S2). The VP0 (VP4/VP2), VP3, and VP1 regions showed phylogenetic clades with 66.1% (39/59), 0% (0/59), and 40.7% (24/59) intersections, respectively. The phylogenetic trees of the 2A, 2B, 2C, and 2BC regions indicated phylogenetic conflicts of 79.7% (47/59), 35.6% (21/59), 57.6% (34/59), and 64.4% (38/59), respectively. The 3A, 3B, 3AB, 3C, 3D, and 3CD regions showed phylogenetic clades with 83.1% (49/59), 83.1% (49/59), 88.1% (52/59), 59.3% (35/59), 74.6% (44/59), and 69.5% (41/59) intersections, respectively. The genotypic tree of the VP3 region had no phylogenetic conflict (100% phylogenetic parallel patterns) compared with the whole-genome tree. Representative trees of the VP1/P2A, VP1/P2B, and entire VP1 regions showed phylogenetic conflicts of 76.3% (45/59), 94.9% (56/59), and 35.6% (21/59), respectively.

## 4. Discussion

The genomic epidemiology of food-borne viruses plays a critical role in identifying and tracking the source of pathogens during outbreaks [35,36,37,38,39,40,41,42]. Partial-genome sequences are suitable for investigating the presence of a virus and its genotypic diversity but not for identifying the geographical origin or tracing back the source of an outbreak. In 1990, WGS was applied for analyzing the molecular epidemiology of a prototype norovirus [26] and revealed the periodic emergence of globally spreading variants as well as the origin of food-borne outbreaks [36]. The WGS of hepatitis E virus revealed the transmission source in infected animals or humans, associated with food intake, such as of contaminated shellfish, salad, or vegetables [35,39]. However, the clinical use of WGS using Sanger sequencing and NGS is limited due to the low levels of viral RNA in human samples [21,43,44,45,46]. Sanger sequencing requires a markedly long time to yield whole-genome sequences from patient samples with ultra-low viral loads. The application of WGS is often limited by the lack of NGS technology and the high diagnostic test cost. However, NGS has revolutionized the acquisition of whole genomes by generating high-yield genomic data from small-concentration samples. For instance, novel NGS methods, such as the shotgun metagenomics-based workflow and target enrichment, were used for identifying SARS-CoV-2 in clinical samples [23,24]. The nanopore sequencing method was used to obtain nearly whole-genome sequences of HAV from cultured cells [25]. However, NGS-based WGS has not yet been attempted for detecting HAV in patient samples [37,46,47]. Here, multiplex PCR-based NGS was developed to ensure high genome coverage of HAV in clinical samples containing a relatively low viral load. Target-enriched NGS is an essential method for successfully obtaining genome sequences of HAV directly from patient samples. Our results suggested that multiplex PCR-based NGS of HAV clinical samples could help investigate the genotypic diversity and tracking of HAV in global outbreaks.

Human-to-human transmission of hepatitis A poses a critical public health threat owing to a high proportion of men who have sex with men (MSM) [48]. In 2015–2020, the widespread outbreaks of hepatitis A among MSM have been reported in Taiwan, Berlin, Netherlands, and United States [49,50,51,52,53,54]. Genomic epidemiology of MSM-associated HAV outbreaks allows to detect the infection origin, transmission chains linked to outbreaks or sporadic cases, and populations at high risk. In phylogenetic studies, whole-genome sequences are required to interpret fundamental phylogenetic and genotypic identification of HAV in humans, as well as animals and plants [24,26,35,39,55]. Whole-genome sequencing of the viruses become increasing overall phylogenetic support, while the phylogeny of partial-genome sequences yielded the incomplete lineage sorting in each genomic region [56,57,58]. The construction of phylogenetic conflict signals may be associated with evolutionary biological mechanisms such as recombination, host-switching and co-speciation events [6,59,60,61,62,63,64,65]. A previous phylogenetic analysis of the hepatitis B virus showed that partial-genome sequences were insufficient for defining phylogeographical links owing to phylogenetic branches with low support, despite identifying new genotypes [56]. The partial-genome with high genomic variability of HAV, such as VP1/P2A and VP1/P2B junctions, has been used to identify and classify HAV genotypes worldwide [38,41,42,46,57,66]. Here, the accuracy of genotyping to describe the phylogenetic relationship of HAV strains has not been studied using partial-genome sequences. In this study, the partial genomic sequence of VP1/P2A, VP1/P2B, and entire VP1 regions showed 35.6–94.9% phylogenetic conflicts compared to the whole-genome sequences of HAV. These highly variant regions might be useful for categorizing HAV genotypes but not for identifying reliable phylogenetic relationships or infection sources. In contrast, the partial genomic sequence of HAV VP3 region demonstrated a significantly no phylogenetic conflict (100% phylogenetic parallel patterns) compared with that of the whole-genome sequences, indicating that it might be useful for distinguishing viral genotypes and detecting the source of viral infection in emerging HAV outbreaks. Furthermore, the VP3 gene could be used for the rapid molecular diagnosis of hepatitis A by real-time qPCR, as differences in quantification with the consensus VP4 gene. Therefore, these findings would allow the rapid and accurate molecular detection, genotypic identification, and monitoring of emerging HAV. The limitation of our study is the paucity of clinical samples for various HAV strain comparisons, and thus, further research is needed to characterize the VP3 region and evaluate its accuracy in viral surveillance.

## 5. Conclusions

Nearly whole-genome sequences of HAV using multiplex PCR-based NGS facilitate definition of the genetic diversity, molecular epidemiology, and origin of outbreaks. The VP3 gene might serve a representative genetic marker for tracking the infectious origin and molecular diagnosis of emerging HAV outbreaks. Thus, this study provides significant insights into the disease control and preparedness of hepatitis A patients at high risk of severe illness.

## Figures and Tables

**Table 1:** Characteristics and laboratory test results of patients with acute hepatitis A infection in the Republic of Korea.

Observation	HAV KUMC 98-34	HAV KUMC 02-1	HAV KUMC 04-1	HAV KUMC 14-1	HAV KUMC 14-2
Year	1998	2002	2004	2014	2014
Age	28	30	22	38	36
Gender	Male	Male	Female	Female	Female
Anti-HAV IgM	Positive	Positive	Positive	Positive	Positive
Anti-HAV IgG	Negative	Negative	Negative	Negative	Negative
ALT (IU/L)	5431	4672	10,592	2572	456
AST (IU/L)	3447.8	2389	18,912	1872	410
ALP (IU/L)	166	207	184	236	232
Total bilirubin (mg/dl)	4.55	8.00	3.40	3.73	2.98
Prothrombin time (sec)	49	74.2	38.1	89	105
Albumin (g/dl)	3.79	3.63	4.36	3.4	3.1
HBsAg	Negative	Negative	Negative	Negative	Negative
Anti-HCV	Negative	Negative	Negative	Negative	Negative

ALT, alanine aminotransferase; AST, aspartate aminotransferase; ALP, alkaline phosphatase; anti-IgM, anti-immunoglobulin M; anti-IgG, anti-immunoglobulin G; HAV, hepatitis A virus; HBsAg, hepatitis B surface antigen; HCV, hepatitis C virus.

**Table 2:** Summary of total reads and read mapping to hepatitis A virus (HAV) genomes by multiplex polymerase chain reaction-based next-generation sequencing.

HAV RNA Copy Number (log10 copies/μL)	Sample	Sample Type	Ct Value	HAV Coverage ^1^	Total Reads	Reads Mapped to Reference Sequence ^1^	Mapping Reads /Total Reads	Depth of Coverage ^2^
2.2	HAV KUMC 14-1	Serum	27.6	94.6%	3,072,916	2,835,008	92.3%	56,719
1.4	HAV KUMC 14-2	Serum	29.8	94%	4,391,220	2,969,551	67.6%	57,894
1	HAV KUMC 02-1	Stool	31	92.3%	2,030,476	1,382,172	68.1%	27,264
0.3	HAV KUMC 04-1	Serum	33.3	86.1%	4,328,092	861,676	19.9%	16,630

^1.^ Virus coverage and reads mapped to a reference sequence were calculated using the LU38 strain from China. ^2.^ Depth of coverage was calculated by the number of mapped reads (read length × number of reads matching the reference/reference genome size).

**Table 3:** Genetic relationship between hepatitis A virus (HAV) from the Republic of Korea and representative subgroups.

		Nucleotide (%)					Amino Acid (%)				
Genotype	Strain	HAV KUMC 98-34	HAV KUMC 02-1	HAV KUMC 04-1	HAV KUMC 14-1	HAV KUMC 14-2	HAV KUMC 98-34	HAV KUMC 02-1	HAV KUMC 04-1	HAV KUMC 14-1	HAV KUMC 14-2
IA	HAV KUMC 98-34	-	-	-	-	-	-	-	-	-	-
	HAV KUMC 02-1	96.4	-	-	-	-	99.1	-	-	-	-
	HAV KUMC 04-1	97.4	96.2	-	-	-	98.7	98.7	-	-	-
	HAV KUMC 14-1	97.8	95.7	97.6	-	-	99.4	98.8	98.3	-	-
	HAV KUMC 14-2	96.5	95.5	95.7	96	-	99.2	99.1	98.4	98.9	-
	LU38	97.9	96.7	97	97.1	97.4	99.1	99.3	98.5	98.8	99.1
	H2	96.3	97.9	96	95.7	95.4	99.6	99.3	98.7	99.1	99.4
	AH1	96.4	97.8	95.9	95.7	95.6	99.1	99.3	98.5	98.8	99
	HAJFF-Kan12	98.2	96.2	98.2	99.3	96.3	99.7	99.3	98.9	99.4	99.5
	GBM	95.7	95.5	95.1	95.1	95	98.6	98.6	97.9	98.2	98.4
IB	HM-175	91.5	91.3	91.4	91.2	90.6	98.8	98.9	98.1	98.4	98.8
	MBB	91.6	91.3	91.2	91.1	90.8	98.2	98.3	97.5	97.8	98.2
IIA	CF53/Berne	86.1	86.2	85.9	85.9	86	96.5	96.5	95.7	96	96.3
IIB	SLF88	86.5	86.3	86.3	86.4	86.1	97.1	97.3	96.5	96.7	97
IIIA	Kor-HAV-F	82.9	83.4	82.8	82.7	82.5	94.2	94.6	93.6	93.8	94
IIIB	HAJ85-1	83	83.2	82.8	82.8	82.8	94.4	94.5	93.8	93.9	94.1

IA (China: LU38, H2; Japan: AH1, HAJFF-Kan12; Germany: GBM), IB (Australia: HM175; North Africa: MBB), IIA (France: CF53/Berne), IIB (Sierra Leone: SLF88), IIIA (Korea: Kor-HAV-F), and IIIB (Japan: HAJ85-1).

## Data Availability

The datasets generated for this study can be found in Genbank, under accession number MW405346–MW405350.

## Supplementary Materials

The following are available online at https://www.mdpi.com/article/10.3390/microorganisms10010100/s1, Table S1: Primers for whole-genome sequencing of hepatitis A virus. Figure S1: Comparison of nucleotide similarities within various hepatitis A virus (HAV) genome segments at the genotype level. Figure S2: Tanglegram of hepatitis A virus (HAV) sequences based on whole- (left) and partial- (right) genome phylogenies.
