# Supplementary material for: Genotyping and Molecular Diagnosis of Hepatitis A Virus in Human Clinical Samples Using Multiplex PCR-Based Next-Generation Sequencing
Source: Microorganisms. 2022 Jan 4;10(1):100. doi: 10.3390/microorganisms10010100 (PMC8779169; doi:10.3390/microorganisms10010100)
Supplement: Supplementary file 1 [file microorganisms-10-00100-s001.zip › microorganisms-1457695-supplementary.pdf]

Supplementary materials

Table S1. Primers for whole-genome sequencing of hepatitis A virus.

Oligo ID	Sequence (5′ → 3′)	Position
1F	TTCAAGAGGGGTCTCCGGA	1-19
84F	AGGCTATAGGCTAAATTTCCCT	84-105
208F	ACGCTTTCTGTCTCYTTTCTT	208-228
493F	CATTCAACGCCGGAGGACT	493-511
732F	TAATGAATATGTCCAGACAAGGT	732-754
923F	GAACCTTTGAAAACCTCTGTTG	923-944
1426F	TTTACAGATTTGGAGTTGC	1426-1444
1528F	TATGAAGATGCAAGGGCAAA	1528-1547
1818F	TTGATTTYCAGGTTTTTCC	1818-1836
2210F	GGAGATGATTCAGGAGGTTTT	2210-2230
2412F	CTGAATTGAARCCTGGAGAGT	2412-2432
2746F	TTACAAAACTGCCCTTGGA	2746-2764
3263F	ACTGAGGAGCATGAAATAATGAA	3263-3285
3591F	TGTTGCATTGGCTAAATCC	3591-3609
4103F	AATTATGGCAADAAGAAGGAT	4103-4123
4260F	TGAGAACTGTYCAYTCAATGG	4260-4280
4394F	TGTGAGCCAGTTGTTTGCT	4394-4412
4636F	ATGCCCAATGAGATTGAATA	4636-4655
4846F	GGCTAAAACAAATGATGCAATT	4846-4867
5007F	CAGATGATGAYAATGATAGTGC	5007-5028
5674F	GGAGATGTACCTAGAGCCTTG	5674-5694
5788F	AGGTCTTCCWGGAATGTGTG	5788-5807
6082F	AGTCCCATTCATCATCACATTG	6082-6103
6678F	ATTTTTCTGCYTTTGATGCTAGT	6678-6700
6936F	TGTGGTTCAATGCCTTCTG	6836-6854
7027F	GTGCCTCAATTGAAGCCAGT	7027-7046
595R	TGCCCTAAGCACAGAGAGGT	576-595
1093R	AACCTTGAACAGCAAACTGC	1074-1093
1485R	AATTCATTTCTCATCATCTGTGT	1463-1485
1680R	GAATCTGAAGCATTAAATGGA	1660-1680
1998R	ATCCAAGGAACACGAAATCT	1979-1998
2230R	AAAACCTCCTGAATCATCTCC	2210-2230
2379R	GGATCYTCAATTGTTGTAATAGC	2357-2379
2578R	TGGCAAACCATGAGGAGGATTAG	2556-2578
2921R	AATGATTGTAATTTGCAATCT	2901-2921
3285R	TTCATTATTTCATGCTCCTC	3266-3285
3354R	CCAGCAGCCAAAGARAATCCA	3334-3354
3911R	AGATAACTGAGCAGCCAAT	3893-3911
4179R	GCTTCCTCAATYGCTTTCTCTAT	4157-4179
4413R	TAGCAAACAACTGGCTCAC	4395-4413
4763R	CAATKGCTTCCTTAACATAAACT	4741-4763
4917R	AATGAAACATTATGTCCATCCAT	4895-4917
5240R	TCACGCCATGGTAAACCCC	5222-5240
5390R	CTCCTAAGGCATTCATAACCC	5370-5390
5583R	TCTCTAAACTTDGGAATTGTAGGA	5560-5583
5818R	CAAGGCCCCACCACACATT	5800-5818
6364R	GGACATAAGGAAACCCAGG	6346-6364
6560R	AACTAATAGCTGGACCCCAATAC	6538-6560
6674R	ATGCCAATAGCAACACCTGT	6655-6674
6792R	ATAGTRTTGATAAGAGCYGTTCC	6770-6792
7278R	TGCATAAAAGCAAACCACT	7260-7278
7477R	ATTTACTGAWAARARAAATAAACA	7454-7477

F: forward primer; R: reverse primer.

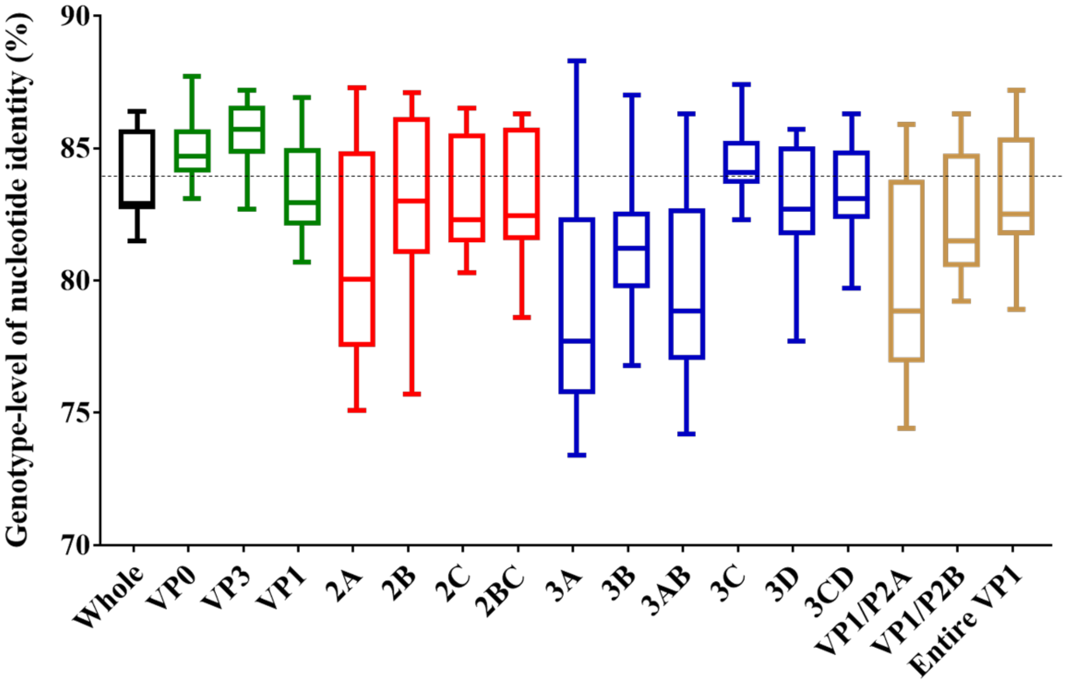

Figure S1. Comparison of nucleotide similarities within various hepatitis A virus (HAV) genome segments at the genotype level. The median value of nucleotide identity is shown by a dashed line. The whole-genome sequences showed 81.8–86.4% nucleotide similarities at the genotype level. The highest sequence similarities were detected in the VP0 (VP4/VP2; 83.1–87.7%), VP3 (82.7–87.2%), and 3C regions (82.3–87.4%), followed by the VP2, VP1, 2A, 2B, 2C, 2BC, 3A, 3B, 3AB, 3D, and 3CD regions (73.4–86.3%). The VP1/P2A, VP1-P2B, and entire VP1 regions exhibited 77.4–87.2% genotype similarities.

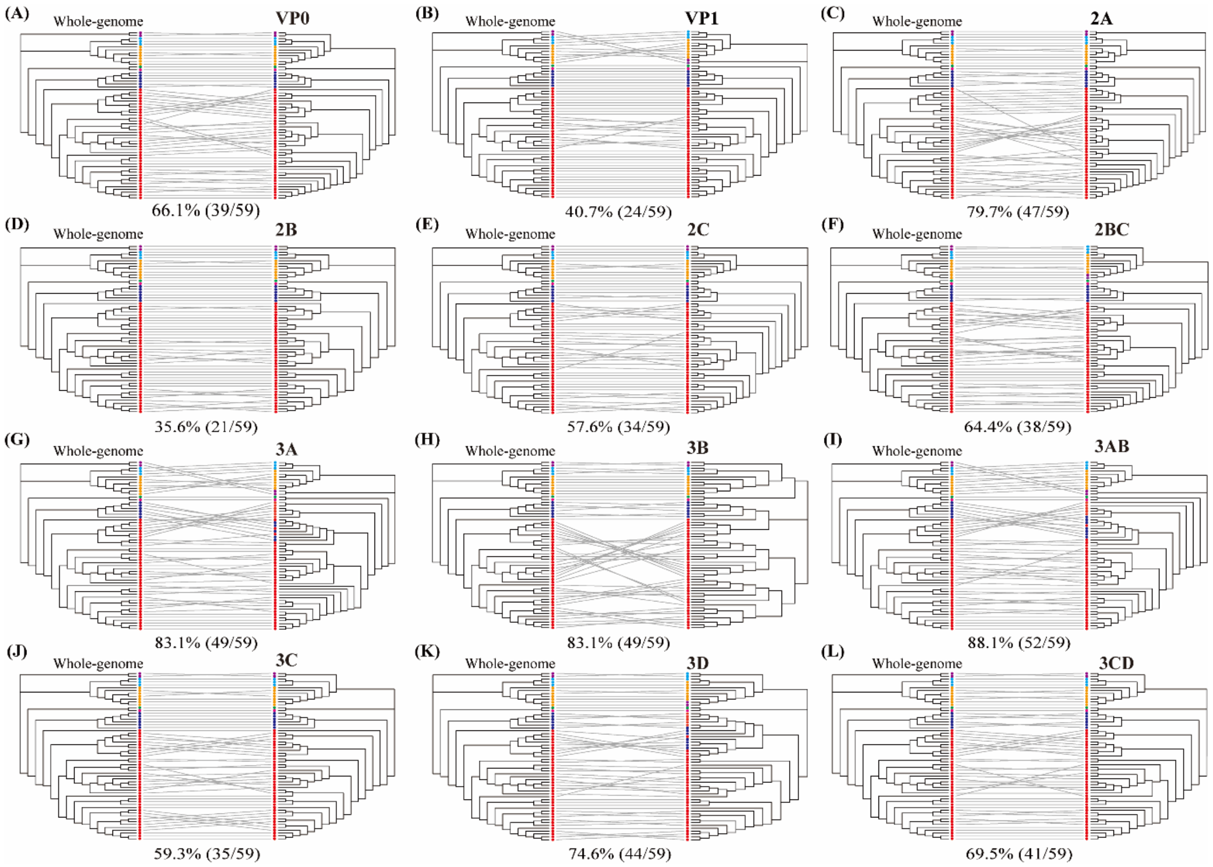

Figure S2. Tanglegram of hepatitis A virus (HAV) sequences based on whole- (left) and partial- (right) genome phylogenies. (**A, B**) VP0 (VP4/VP2) and VP1 in P1 region; (**C–F**) 2A, 2B, 2C, and 2BC in P2 region; (**G-L**) 3A, 3B,

3AB, 3C, 3D, and 3CD in P3 region. Whole-genome and partial-genome phylogenies were generated using the maximum likelihood method. The full lines indicate significant phylogenetic conflicts. Gray lines indicate phylogenetic clade patterns falling between two different regions. The phylogenetic trees of 12 partial-genome sequences showed 35.6–88.1% various phylogenetic conflicts compared with the whole-genome tree.
